# Protocol for a Delphi Consensus Study on Transitional Cognitive Decline in Alzheimer's Disease

**DOI:** 10.1002/alz70857_106674

**Published:** 2025-12-25

**Authors:** Andrew M. Kiselica, Leslie S. Gaynor, Kelly J. Atkins, Dustin B. Hammers, Kevin Duff

**Affiliations:** ^1^ University of Georgia, Athens, GA, USA; ^2^ Division of Geriatric Medicine, Department of Medicine, Vanderbilt University Medical Center, Nashville, TN, USA; ^3^ Vanderbilt University Medical Center, Nashville, TN, USA; ^4^ Memory and Aging Center, University of California San Francisco, San Francisco, CA, USA; ^5^ The University of Melbourne, Melbourne, VIC, Australia; ^6^ Indiana University School of Medicine, Indianapolis, IN, USA; ^7^ Oregon Health & Science University, Portland, OR, USA; ^8^ NIA‐Layton Aging & Alzheimer's Disease Research Center, Portland, OR, USA

## Abstract

**Background:**

An Alzheimer's Association workgroup published new clinical criteria for the diagnosis and staging of Alzheimer's disease (AD) that includes a six‐stage (1‐6) clinical staging schema. Stages 1 (asymptomatic stage) and 3‐6 (mild cognitive impairment and dementia stages) are familiar to clinicians.

However, Stage 2 does not have a known clinical analogue; it consists of biomarker positivity and transitional cognitive decline (TCD) that does not rise to the level of impairment. To ensure we capture people in Stage 2, we need consensus on the nomenclature and measurement strategies used to define TCD. This goal is especially important given the advent of new AD criteria and the increasing number of clinical trials of pharmacotherapies targeting early stages of AD. This presentation will outline a protocol for conducting a Delphi study to establish consensus regarding the nomenclature and measurement of TCD in AD.

**Method:**

Leaders from the International Neuropsychological Society Dementia Special Interest Group are convening a steering committee of ∼10 individuals with expertise in TCD, Delphi studies, and mixed methods research. The steering committee will guide the conduct of a Delphi consensus study, including identifying Delphi panel members, developing and disseminating surveys, and analyzing and summarizing data on consensus.

**Result:**

We propose the following protocol for the Delphi panel study. 1. Delphi panel selection: Identification of ∼30 Delphi panel members via majority vote of steering committee members or selected from among authors identified in a literature review on TCD. 2. Survey development: Develop ∼30 survey items focused on the most appropriate clinical label for TCD and the most promising measurement approaches for this construct. 3. Data collection: Collect participant responses via online surveys over 3 iterative rounds with summary feedback provided to participants between rounds. 4. Data analysis: Consensus to be defined by 75% agreement among Delphi panelists, with qualitative feedback assessed to provide insight into factors promoting or preventing consensus.

**Conclusion:**

Completing this Delphi study will provide consensus‐based resources to inform clinical trial selection and analytic processes for interventions targeting the earliest stages of AD.

